# Polymer–Silicate Composite Gel Systems for Enhanced Chloride Resistance of Cement-Based Materials

**DOI:** 10.3390/gels11120936

**Published:** 2025-11-21

**Authors:** Tianhang Zhang, Yonggui Dai, Shuai Ren, Zhengqiang Huang, Chong Han, Wencheng Ding

**Affiliations:** 1 School of Water Conservancy and Transportation, Zhengzhou University, Zhengzhou 450001, China; daiyg@gs.zzu.edu.cn (Y.D.); rs301077@163.com (S.R.); hanchong@gs.zzu.edu.cn (C.H.); dwc4113@gs.zzu.edu.cn (W.D.); 2 Zhongyuan Research Institute, Zhejiang University, Zhengzhou 450000, China; hzq124@gmail.com; 3 Henan Province Engineering Technology Research Center of Infrastructure Disease Monitoring and Treatment, Zhengzhou 450000, China

**Keywords:** polymer–silicate composite gel system, silane, fluorocarbon, chloride ion resistance

## Abstract

To address the issues of insufficient protection and poor durability in concrete during service, this study developed a novel polymer–silicate composite gel system by combining silane with fluorocarbon resin emulsion and applied it to mortar specimens. The chloride ion resistance enhancement of mortar provided by the novel gel system was evaluated using the RCM method and natural chloride ion penetration tests, with SEM images employed to analyze its anti-permeation mechanism. Results indicate that the chloride ion migration coefficient of the novel composite gel system is 4.91 × 10^−12^ m^2^/s, representing a 63.97% reduction compared to the single fluorocarbon gel system. Within the 0–5 mm depth range, free chloride ion contents at 14, 28, and 56 days decreased by 55.35%, 50.10%, and 43.64%, respectively, demonstrating excellent resistance to chloride penetration. Acid and alkali resistance tests demonstrated that the system retained the inherent corrosion resistance of the fluorocarbon component. Carbonation tests demonstrated that the system exhibited a slight decrease in carbonation resistance compared with the pure fluorocarbon gel system, while still maintaining a satisfactory performance level. Overall, the polymer-silicate composite gel system significantly enhanced the mortar’s resistance to chloride ion penetration.

## 1. Introduction

Concrete, a typical cement-based gel material, is highly susceptible to attack by harmful ions due to surface pores and cracks, particularly in marine, chemical, and saline-alkali environments, where acid and alkali ions can severely impact its structure [1,2,3]. Excessive concentrations of Cl^−^, SO_4_^2−^, and OH^−^ compromise the calcium silicate hydrate (C-S-H) gel, resulting in severe structural damage. Moreover, anions can infiltrate the interior via diffusion, permeation, and capillary absorption, ultimately inducing steel corrosion [4,5,6,7]. A practical and effective way to enhance the durability of concrete is the application of surface protective materials [8]. Currently, surface protection for concrete mainly relies on polymer emulsions and silane-based coupling agents [9,10]. Polymer emulsions include epoxy, acrylic, polyurethane, fluorocarbon, and other types. These protective materials form a polymer-silicate gel system with concrete, creating a dense protective layer on the surface that effectively hinders water molecules and harmful ions introduced by water from penetrating the matrix [11,12,13]. Silane coupling agents mainly consist of silanes and siloxanes. These materials can penetrate into the concrete structure and react with C-S-H gel in the concrete to form a hydrophobic layer, preventing the penetration of water molecules and various harmful ions [14,15]. Numerous studies have confirmed that both types of protective materials can effectively improve the durability of concrete.

Almusallam et al. [16] compared five types of polymer emulsions—polyurethane, epoxy resin, chlorinated rubber, acrylic, and polyethylene—on improving the durability of concrete. The results indicated that all these polymers significantly reduced the penetration of water and chloride ions, with epoxy and polyurethane being most effective in enhancing substrate hydrophobicity. Vipulanandan et al. [17] evaluated the performance of two polyurethanes in a sulfuric acid environment and found that both effectively prolonged the service life of concrete. Merah et al. [18] compared acrylic and epoxy for carbonation resistance, revealing that epoxy more effectively decreased the carbonation depth of the matrix. Zheng et al. [19] evaluated the effects of waterborne fluorocarbon resin and silicone-acrylic emulsions on the water absorption and chloride ion resistance of concrete. The findings demonstrated a marked enhancement in chloride ion resistance and a significant decrease in water absorption. However, epoxy resins have poor toughness and are susceptible to aging [20]. Polyurethane exhibits excellent corrosion resistance, but its application demands high technical standards and it has relatively poor thermal stability [21]. Acrylics possess good weather and UV resistance but show poor water and chemical corrosion resistance, making them unsuitable for marine environments [15]. Fluorocarbons offer excellent weather and corrosion resistance, but their high rigidity makes them prone to cracking and peeling [22,23]. Although polymer-silicate gel systems can effectively enhance the matrix’s durability, they have inherent drawbacks that hinder long-term structural protection.

Silane coupling agents have received wide attention due to their film-forming penetration properties, and their applications in concrete mainly focus on silanes and their derivatives [24,25]. Studies have shown that silanes can penetrate the micro-pores of concrete and, through de-alcoholization reactions with water as well as chemical reactions with hydroxyl groups in the concrete, form a three-dimensional cross-linked network of organosiloxane resin. This network achieves tight bonding with the C-S-H gel in the structure, preventing the ingress of water and various harmful ions [26,27,28]. Zhu et al. [29] systematically investigated the anti-corrosion performance of silanes in offshore concrete structures. Their results indicated that silanes could penetrate 2–3 mm into the surface layer of the structure, and silane-impregnated concrete exhibited significantly reduced water absorption and chloride ion uptake. Li et al. [30] incorporated different amounts of silanes into cement-based materials. Experiments demonstrated that silanes enhance crack resistance, increase contact angle, and improve impermeability, while reducing capillary water absorption, thereby generally strengthening water resistance. SEM and nitrogen adsorption tests revealed that the silane–silicate gel system exhibited higher maximum adsorption capacity and total pore volume compared to the control group, indicating that the silane component generated hydrophobic silicone resin within the pore network of the C–S–H gel in the matrix, converting a large number of internal pore surfaces into hydrophobic interfaces. However, after penetrating into the interior of the concrete, the hydrophobic layer formed by silanes only coats the surfaces of the internal pores without sealing them and cannot establish a dense protective layer on the concrete surface. Such materials are limited in enhancing carbonation resistance and weather durability [31].

The limitations of polymer–silicate gel systems are closely associated with the intrinsic structure of the cementitious substrate. The protective layer formed by the polymer emulsion effectively mitigates the detrimental effects of ultraviolet radiation, rainfall, and other environmental exposures on the substrate. However, concrete, as a highly porous material, contains water molecules and various mobile ions within its pore network, which can adversely influence the structure and durability of the film-forming coating. Fluctuations in ambient temperature and humidity cause volumetric expansion of internal moisture and gases, generating pressure on the protective film and eventually leading to reduced adhesion or even cracking. In addition, aggressive ions—particularly chlorides—react with other species during migration to form crystalline products, inducing volumetric expansion and microcracking that accelerate surface degradation and compromise the long-term durability of the coating. To overcome the inherent limitation of polymer–silicate gel systems in addressing the internal pore structure of concrete, numerous studies have been conducted. For instance, the incorporation of fluoroalkyl chains (–(CH_2_)_n_CF_3_ or –(CF_2_)_n_–) into silane molecules has been shown to markedly reduce surface energy and enhance the overall weatherability of the system [32,33,34]. Silane-functionalized nano-silica effectively enhances coating hydrophobicity and weather resistance, thereby reducing chloride ion ingress [35,36]. However, although such modified silane systems can improve the internal pores of concrete and reduce the impact of water, oil, or ultraviolet radiation on the surface of the structure, they do not enhance carbonation resistance, and therefore the service life of the protective system remains limited. Some researchers have attempted to incorporate silane into polymer–silicate gel systems. Huang et al. [37] reported that silane-modified acrylic resin coatings improved interfacial bonding, hydrophobicity, and UV aging resistance, while Pramualkijja et al. [38] demonstrated that siloxane-modified polyacrylate coatings exhibited superior hydrophobicity, self-cleaning properties, and interfacial adhesion. Although these modifications enhance interfacial adhesion, they involve complex and costly procedures and fail to adequately address the potential deterioration induced by ion migration within the concrete or the synergistic corrosion mechanisms arising from both internal and external environments.

In this study, a novel polymer–silicate composite gel system was developed to protect concrete by integrating silane and fluorocarbon components. Compared with the previously mentioned modified silane coatings, this system preserves the excellent weathering and corrosion resistance of fluorocarbon coatings while overcoming the durability limitations of purely penetrating coatings. Compared with chemically silane-modified polymer coatings, the silane component in this system can penetrate the internal pores of the concrete and coat their surfaces, effectively preventing potential damage to the surface layer caused by the migration of harmful ions within the concrete, and it can be easily prepared through simple mechanical mixing. This study systematically evaluated the chloride ion protection performance of a silane-modified fluorocarbon–silicate composite gel system and analyzed the substrate’s microstructure at the microscopic level using scanning electron microscopy. The vulnerability of single-film coatings to internal and external harmful ions was examined, and the synergistic protective mechanisms of the composite system were investigated. Furthermore, the effects of strong acidic and alkaline environments on the substrate were assessed to determine its corrosion resistance under harsh conditions, and carbonation tests were conducted to compare the carbonation resistance of different systems. This work provides important theoretical insights into the degradation mechanisms of concrete coatings and offers a solid foundation for the practical application of this novel protective system in concrete structures.

## 2. Results and Discussion

### 2.1. Chloride Ion Migration Coefficient

**Figure 1:**
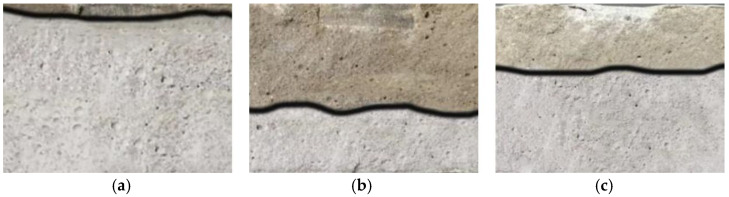
Measured depth of chloride penetration of mortar specimens: (**a**) Blank; (**b**) SF/Si; (**c**) F/Si.

Figure 1 presents the measured chloride ion penetration depths from the RCM test, where the silvery-white regions indicate the extent of chloride penetration. Specifically, Figure 1a shows the blank control group, Figure 1b represents the silane/fluorocarbon-silicate composite gel system (hereafter SF/Si), and Figure 1c depicts the fluorocarbon-silicate gel system (hereafter F/Si). It is evident that both systems exhibit markedly lower chloride ion penetration depths than the blank control group. The penetration depth of SF/Si is reduced by 57% relative to F/Si, demonstrating that silane modification substantially improves the coating’s impermeability. Furthermore, hydrophobic layer traces formed by silane infiltration are visible on both the side and top surfaces of SF/Si, offering stronger protective performance than F/Si.

Table 1 presents the chloride ion migration coefficients of the two gel systems. Statistical analyses were first performed using Levene’s test to verify homogeneity of variance (*p* > 0.05). One-way ANOVA was then applied to assess the effect of different systems on chloride penetration depth and DRCM values, revealing statistically significant differences (*p* < 0.05). To further identify which groups differed, Tukey HSD post-hoc tests were conducted. The results confirmed that the silane-modified fluorocarbon–silicate gel system exhibited a significantly lower chloride penetration coefficient compared with the pure fluorocarbon system (*p* < 0.05). These analyses demonstrate that variations in system composition are the primary factor responsible for the observed differences in chloride resistance. Compared with the blank control, the chloride ion migration coefficients of SF/Si and F/Si were significantly reduced, with DRCM values decreasing by 88.1% and 67.1%, respectively. The D_RCM_ value of SF/Si was 63.97% lower than that of F/Si, indicating that silane significantly enhances the fluorocarbon’s barrier effect against chloride ions, exerting a stronger restriction on their migration. The D_RCM_ value of SF/Si was 63.97% lower than that of F/Si, indicating that silane modification greatly enhanced the fluorocarbon’s chloride ion barrier properties, effectively restricting chloride ion migration. As a surface film-forming coating, fluorocarbon blocks the pores of the mortar surface, isolating it from external chloride ions and forming an effective chloride ion barrier. However, its protection is limited to the surface; once the barrier is penetrated, chloride ion infiltration into the mortar follows a similar pattern to that in the untreated control, thereby restricting its long-term protective capacity. Chloride ion penetration is usually accompanied by water molecule movement. Silane molecules penetrate the specimen, where they hydrolyze to form silanol, which subsequently reacts with hydroxyl groups in silicates to generate siloxane chains. These siloxane structures condense and deposit onto the mortar pore surfaces, forming a hydrophobic silicone resin layer that effectively impedes chloride ion migration [39,40]. Therefore, adding a small amount of silane emulsion enhances the hydrophobicity of the fluorocarbon component, thereby improving the system’s resistance to chloride ion penetration. The silane/fluorocarbon-silicate composite gel system demonstrates both the sealing effect of the fluorocarbon and the hydrophobic effect of the silane. The synergistic interaction between silane and fluorocarbon not only decreases the chloride ion migration coefficient but also enhances the durability of the mortar.

**Figure 2:**
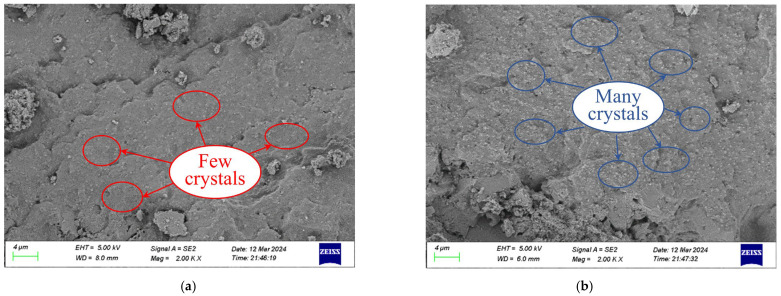
SEM images of the two gel systems after undergoing the RCM test: (**a**) SF/Si; (**b**) F/Si.

Figure 2 shows the SEM images of SF/Si and F/Si after the RCM test. The images show that NaCl crystals are present in the membrane structures of both SF/Si and F/Si. This phenomenon indicates that during chloride ion penetration, both membrane structures have developed varying degrees of penetration pathways. The amount of NaCl crystals in the structure reflects the extent of membrane penetration. It can be observed that the number of NaCl crystals in Figure 2b is significantly greater than in Figure 2a. This indicates that SF/Si exhibits higher impermeability compared to F/Si, making it more difficult for chloride ions to penetrate. This observation is consistent with the RCM test results.

### 2.2. Chloride Ion Content Distribution

**Figure 3:**
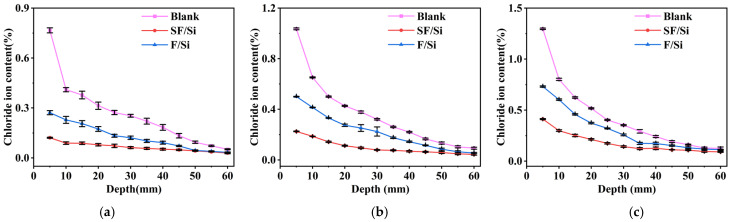
Distribution pattern of internal chloride ion content of mortar under the same soaking time: (**a**) 14d; (**b**) 28d; (**c**) 56d.

The distribution pattern of chloride ion content in mortar under the same immersion time is shown in Figure 3. The chloride ion content is highest within the depth range of 0–5 mm, as the surface layer of the mortar is relatively looser than the interior. This results in lower penetration resistance for chloride ions, causing them to accumulate in the superficial layer and form a high-concentration region. Levene’s test was performed to verify the homogeneity of variance (*p* > 0.05). A two-way ANOVA was conducted to assess the effects of coating system type and immersion time on free chloride ion content. The ANOVA results indicated a significant main effect of coating system type (*p* < 0.05). Subsequently, a simple effects analysis (Tukey HSD) was performed to compare the specific differences among coating systems at each immersion time. The results confirmed that the silane-modified fluorocarbon coating significantly reduced free chloride ion content compared with other systems (*p* < 0.05), supporting the enhanced chloride resistance of the proposed coating. Compared with the blank control group, the free chloride ion content in SF/Si decreased by 84.22%, 78.28%, and 68.23% after 14, 28, and 56 days of immersion, respectively, while that in F/Si decreased by 64.67%, 51.64%, and 43.64%, respectively. Compared with F/Si, the free chloride ion content in SF/Si decreased by 55.35%, 50.10%, and 43.64%, respectively. F/Si forms a dense protective layer between the chloride salt environment and the test block. However, due to its poor hydrophobicity, a small number of chloride ion penetration pathways may form within the protective layer. SF/Si integrates the impermeability of silane and the density of fluorocarbon to form a comprehensive protective system. The silane dispersed within the coating layer reduces the formation of penetration pathways through its intrinsic hydrophobicity, limiting both the inward penetration and lateral migration of chloride ions that have reached the surface layer. Consequently, its resistance to chloride ion penetration is superior to that of F/Si.

**Figure 4:**
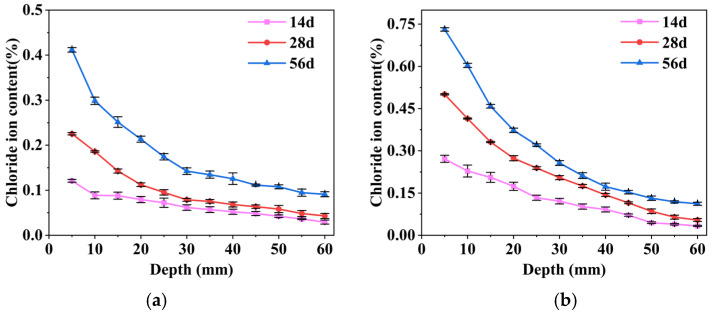
Change rule of internal chloride ion content of mortar under different soaking times: (**a**) SF/Si; (**b**) F/Si.

Figure 4 shows the variation of free chloride ion content in mortar for each coating system under different immersion times. Two-way ANOVA results indicated a significant interaction between coating system and immersion time (*p* < 0.05), demonstrating that the temporal variation of chloride ion content differed significantly among the coating systems. To further investigate specific differences, simple effects analyses (Tukey HSD) were conducted at each immersion time to compare coating systems. The results indicated that, overall, the SF/Si coating consistently exhibited significantly lower chloride ion content than the F/Si coating and the uncoated control at all time points (*p* < 0.05). Overall, the chloride ion concentration in the F/Si system fluctuated more noticeably over time compared to SF/Si. Meanwhile, by comparing the chloride ion content at a depth of 5–15 mm in Figure 4a,b, it can be observed that at a depth of 5–10 mm, the chloride ion concentration in both materials decreases significantly. However, at a depth of 10–15 mm, the rate of decline in chloride ion concentration for SF/Si slows down, especially at 14 days, where there is almost no reduction in chloride ion concentration within this depth range. In contrast, the decline in chloride ion concentration for F/Si at 10–15 mm remains nearly the same as at 5–10 mm, indicating that the reduction in chloride ion concentration in SF/Si slows earlier than in F/Si. This distribution pattern indicates that most chloride ions penetrating into the test block in SF/Si are confined to its surface layer, while F/Si, lacking an internal barrier, allows a greater accumulation of chloride ions in the deeper layers of the test block. This further demonstrates the barrier effect of silane against chloride ions.

**Figure 5:**
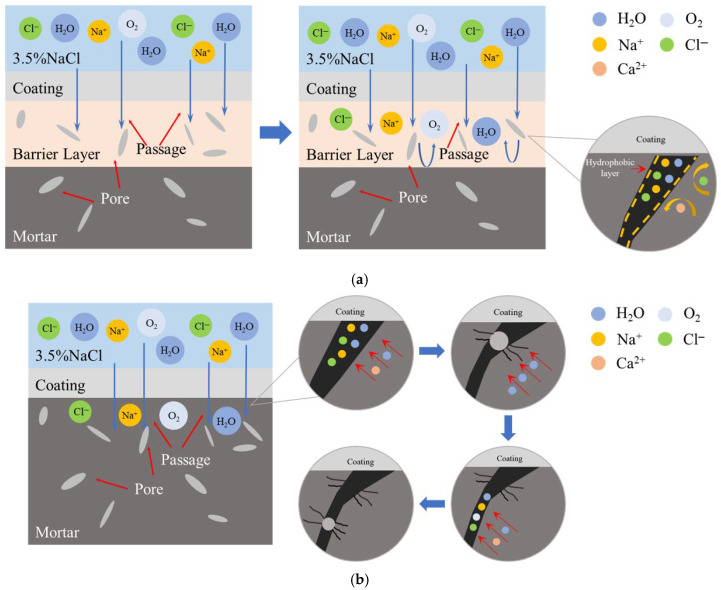
Schematic diagram of the mechanism of chloride ion impermeability of the two systems: (**a**) Mechanism of silane barrier to chloride ions; (**b**) Pore crystallization damage process of F/Si.

The chloride ion penetration resistance mechanism of the two gel systems is shown in Figure 5. Since the protective effect of F/Si is mainly concentrated on the mortar surface, it cannot effectively restrict the inward diffusion of chloride ions passing through the membrane structure. The silane components in SF/Si can penetrate into the test block, forming a barrier zone, which slows the intrusion of water molecules and chloride ions, resulting in a high chloride ion concentration on the test block surface, while the internal chloride ion concentration is significantly reduced [39].

The barrier zone created by silane not only mitigates the penetration of external water molecules and chloride ions but also acts as an internal seal, preventing the migration of chloride ions and water molecules within the mortar, thereby offering internal protection. As mortar inherently contains some moisture, chloride ions migrate freely through its pores, using water as a carrier. During migration, these ions interact with internal Ca^2+^ and Na^+^ ions, precipitating as crystals. Table 2 presents the size data of typical ions and crystals in cement-based materials. The unit cell, as the basic geometric unit of the crystal structure, has a parameter *a* representing one edge length, which can be used to reflect the specific size of the unit cell. According to the data in the table, the unit cell parameter *a* of calcium carbonate is 5 times the radius of the calcium ion and 2.8 times the radius of the carbonate ion. For sodium chloride, the unit cell parameter *a* is 3.11 times the radius of the chloride ion and 5.5 times the radius of the sodium ion. The ionic radius is much smaller than the unit cell parameter of the corresponding crystal, indicating that significant volume expansion occurs during the process of ions combining to form crystals. The mortar is damaged due to the volume expansion caused by the transformation of ions into crystals within its pores. Meanwhile, ions continue to migrate via water molecules into other pores, crystallizing and inducing further damage, as shown in Figure 5b. This crystallization-induced deterioration, similar to freeze-thaw cycles, compromises the integrity of the concrete. If crystallization occurs within the pores near the pores at the material–matrix interface, it weakens adhesion and undermines the durability of the coating [41]. In Figure 5a, the barrier zone formed by silane can block internal ions near the mortar surface, preventing freeze-thaw cycle-like crystallization damage inside the mortar. While enhancing the mortar’s resistance to chloride ions, it also improves its durability.

**Figure 6:**
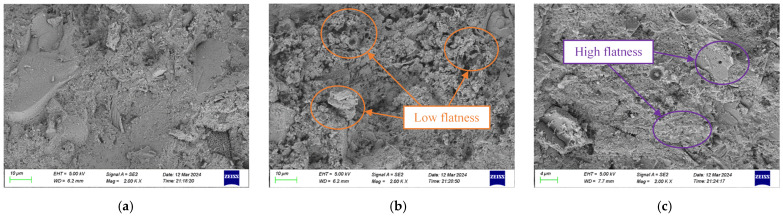
SEM images after 56 d of natural erosion by chloride ions: (**a**) Blank; (**b**) SF/Si; (**c**) F/Si.

The SEM images of chloride ion natural erosion after 56 days for the blank control group, SF/Si, and F/Si are shown in Figure 6. As shown in Figure 6a, the internal structure of the blank mortar is relatively dense, mainly due to the formation of abundant hydration products during the soaking process, which reduces the surface porosity of the mortar. This trend is consistent with the observation that the increase in chloride ion content slows down as soaking time increases [41]. As shown in Figure 6b,c, the surface of F/Si is smoother than that of SF/Si, due to the hydrophobicity provided by the silane molecules, which slowed down the hydration reaction at the silane-impregnated layer of the specimen. This resulted in SF/Si having a lower density compared to F/Si. The effect of SF/Si on the chloride ion resistance of the mortar can be attributed to its permeability resistance. Compared to F/Si, SF/Si has a more significant waterproof effect and can effectively prevent chloride ion penetration, indicating better chloride ion resistance.

### 2.3. Mass Loss Under Acid and Alkali Corrosion

**Figure 7:**
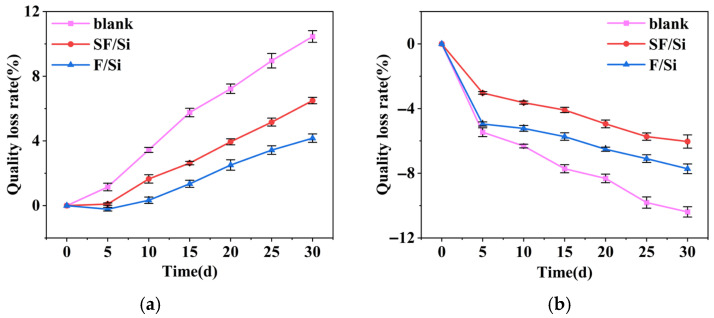
Mass loss rate curves of mortar under acid and alkali exposure: (**a**) Acid exposure, (**b**) Alkali exposure.

The mass loss rate of mortar after acid and alkali exposure indicates its resistance to corrosion. Figure 7 shows the mass loss rates of mortars under different gel systems [42,43]. To evaluate the protective performance of different coating systems, Levene’s test and two-way ANOVA were conducted on the mass loss rate. The results of Levene’s test indicated that the data satisfied the assumption of homogeneity of variance (*p* > 0.05). The two-way ANOVA revealed that the coating system had a significant main effect on the mass loss rate under acidic and alkaline corrosion conditions (*p* < 0.05). The Tukey HSD post hoc test further showed that the differences between the coated groups and the blank control group were statistically significant (*p* < 0.05).

As shown in Figure 7a, SF/Si and F/Si exhibit significantly lower mass loss rates under acid erosion compared to the untreated control. Due to direct exposure to the acidic environment, the untreated control shows a linear increase in mass loss, exceeding 10% after 30 days of acid attack. The high content of F–C bonds in SF/Si and F/Si enables them to largely retain their protective properties in acidic environments, with mass loss rates of approximately 6% and 4%, respectively, after 30 days of acid exposure. The addition of silane emulsion reduces the original compactness of the fluorocarbon film in SF/Si, resulting in a mass loss rate approximately 2% higher than that of F/Si.

As shown in Figure 7b, all mortar samples exhibit negative mass loss rates, with the untreated control showing a substantially higher mass gain than the two mortars. At this stage, the change in mortar mass mainly depends on water absorption and the formation of internal gel products. Due to the absence of a protective coating, the untreated control exhibits higher internal moisture content and more abundant gel formation. In addition, the lower water absorption of SF/Si results in reduced internal moisture and less gel formation compared to F/Si. Consequently, under alkaline attack, SF/Si shows a slightly lower mass change rate than F/Si.

### 2.4. Compressive Strength Loss Under Acid and Alkali Corrosion

**Figure 8:**
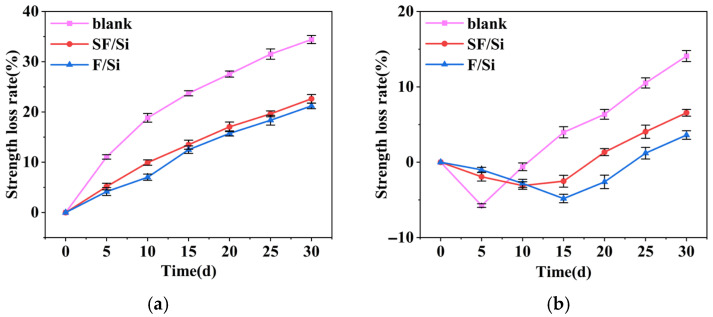
Variation curves of compressive strength loss rate of mortar: (**a**) Acid exposure; (**b**) Alkali exposure.

Figure 8 illustrates the relationship between the compressive strength loss rate of mortars and corrosion time for the two gel systems. Differences among coating systems were evaluated using Levene’s test and two-way ANOVA. Levene’s test confirmed that the compressive strength loss data under both acidic and alkaline conditions satisfied the assumption of homogeneity of variance (*p* > 0.05). The two-way ANOVA indicated that both the coating system type and corrosion time had significant main effects on the compressive strength loss rate (*p* < 0.05). Tukey HSD post hoc tests further revealed that the compressive strength loss in both coating groups was significantly lower than that of the blank control group at all tested time points (*p* < 0.05).

As shown in Figure 8a, under acid attack, the compressive strength loss of all mortars increased progressively over time. At the same exposure duration, the loss rates of SF/Si and F/Si were considerably lower than those of the blank group. Throughout the acid erosion process, the compressive strength loss rates of SF/Si and F/Si were nearly identical, differing by only 1–2%. Both gel systems markedly slowed the early-stage strength loss during acid erosion. The blank group exceeded a 20% loss after 15 days, whereas SF/Si and F/Si reached the same level only after 30 days. This demonstrates their superior acid resistance, with only minor differences between the two.

As shown in Figure 8b, the compressive strength of all mortars subjected to alkaline corrosion initially increased and then decreased. The time at which peak strength was reached differed among the coating systems, further confirming a significant time-dependent effect of coating type (two-way ANOVA, *p* < 0.05). Post-hoc Tukey HSD tests indicated that the compressive strength loss rate of the coated groups was significantly lower than that of the control group (*p* < 0.05), demonstrating that the coating systems effectively enhanced the compressive performance of mortar under alkaline corrosion. At 5 days of alkali exposure, the blank group exhibited a higher strength increase than SF/Si and F/Si. This was due to the higher OH^−^ concentration in the blank mortar, which generated more gel products and enhanced its compactness [44,45]. This observation already reflected the superior alkali resistance of the coated mortars. The strength peaks of SF/Si and F/Si were delayed to around 15 days, further indicating that OH^−^ intrusion into the mortar was effectively mitigated. Beyond 5 days of alkali exposure, the excessive formation of gel products disrupted the internal structure of the mortars. The strength loss rate of the blank mortar increased continuously, far surpassing that of SF/Si and F/Si, while the latter two still exhibited rising strength, further confirming their superior alkali resistance. At 30 days of alkali exposure, the strength loss rate of the blank mortar approached 15%. The compressive strength loss rate of SF/Si remained around 5%, differing from F/Si by only about 2%. This indicates that the incorporation of silane had little influence on the alkali resistance of the fluorocarbon–silicate gel system. In summary, under both acidic and alkaline environments, SF/Si exhibited comparable protective performance to F/Si, suggesting that silane had minimal influence on the acid and alkali resistance of the fluorocarbon–silicate gel system.

In summary, under both acidic and alkaline environments, SF/Si exhibited comparable protective performance to F/Si, suggesting that silane had minimal influence on the acid and alkali resistance of the fluorocarbon–silicate gel system.

### 2.5. Carbonation Depth

**Figure 9:**
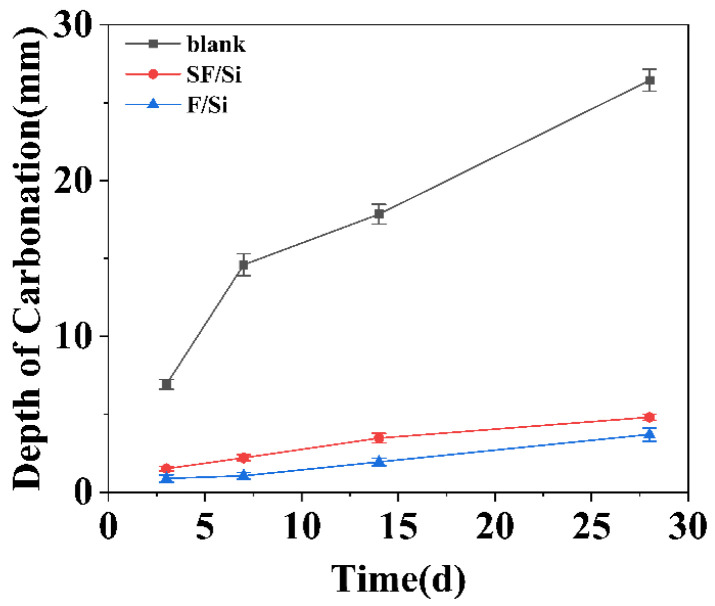
Carbonation depth of mortar at different curing ages.

As indicated by the preceding results, the SF/Si coating exhibited outstanding resistance to chloride ion ingress. Likewise, carbonation resistance represents another critical parameter governing the durability of concrete structures [46]. To further assess the barrier efficiency of the coating against CO_2_ ingress, an accelerated carbonation test was performed. The carbonation depths of the mortars at various curing ages are presented in Figure 9.

Essentially, carbonation involves the diffusion of CO_2_ gas through the coating into the concrete substrate, where chemical reactions occur; the resulting carbonation depth effectively reflects the coating’s protective capability against CO_2_ ingress [47,48]. To examine the statistical significance of inter-system differences, the carbonation depth data were analyzed using Levene’s test for homogeneity of variance, one-way ANOVA, and the Tukey HSD post-hoc comparison. The Levene test confirmed that the data satisfied the assumption of homogeneity of variance (*p* > 0.05). The one-way ANOVA revealed a statistically significant influence of protective system type on the carbonation depth (*p* < 0.05). Subsequent Tukey HSD post-hoc analysis demonstrated that the SF/Si and F/Si coating systems exhibited significantly different carbonation performance compared with the uncoated control (*p* < 0.05).

As illustrated in Figure 9, both protective systems markedly reduced the carbonation depth of the mortars at identical carbonation ages compared with the uncoated control, indicating a pronounced enhancement in carbonation resistance. After 28 days of carbonation exposure, the carbonation depth reached 26.44 mm for the uncoated control, whereas the SF/Si and F/Si systems exhibited depths of 4.82 mm and 3.71 mm, representing reductions of approximately 82% and 86%, respectively, relative to the control. In terms of carbonation depth, the order followed F/Si < SF/Si << uncoated control. According to the Standard for Inspection and Evaluation of Concrete Durability (JGJ/T193-2009) [49], the SF/Si coating exhibited a carbonation depth of 4.82 mm after 28 days of accelerated carbonation testing, corresponding to a carbonation resistance rating of Grade T-IV, which denotes a good performance level, as shown in Table 3. Although the carbonation protection of SF/Si was slightly inferior to that of F/Si, it still satisfied the standard requirements.

Compared with the F/Si system, the SF/Si coating exhibited a marginal reduction in carbonation resistance. This difference is presumed to originate primarily from the limited compatibility between the silane component and the fluorocarbon resin. During the film-formation process, interfacial discontinuities or microdefects may have developed between the two phases, providing additional pathways for CO_2_ diffusion and consequently diminishing the overall gas-barrier efficiency of the coating.

**Figure 10:**
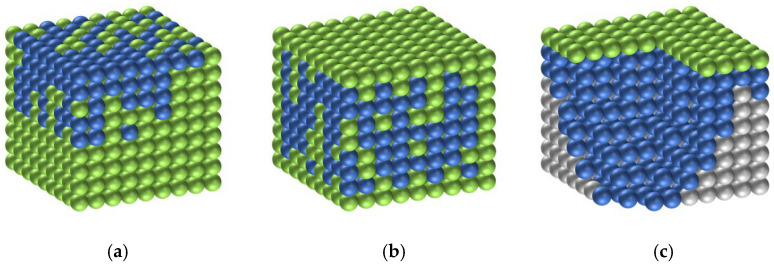
Form of silane emulsion in the SF/Si system: (**a**) Silane components floated on the fluorocarbon surface; (**b**) Silane components embedded within the fluorocarbon matrix; (**c**) Silane components deposited beneath the fluorocarbon coating. Blue spheres represent the fluorocarbon component, green spheres represent the silane component, and gray spheres correspond to the mortar specimens.

As illustrated in Figure 10, a distribution model of silane within the modified protective system was proposed to elucidate its influence on the coating densification and carbonation resistance. Silane species distributed on the coating surface tend to disrupt the continuity of the fluorocarbon film, which primarily accounts for the slight reduction in coating densification and carbonation resistance. In contrast, silane molecules embedded within the coating matrix and deposited near the substrate interface can react with the cementitious phase to form a siloxane network, thereby establishing the dual protective structure proposed in this study, which serves as an essential mechanism underlying the superior chloride-ion impermeability of the composite coating.

**Figure 11:**
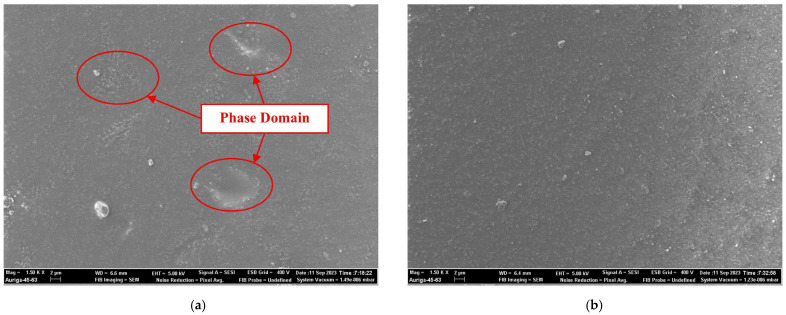
Surface micro-morphology of the two protective systems: (**a**) SF/Si; (**b**) F/Si.

Subsequent SEM observations further validated the proposed model. Figure 11 displays the surface morphologies of the two protective systems. As shown, the F/Si coating developed a continuous and uniform dense film. The region highlighted in Figure 11a corresponds to the phase associated with the silane component. In the SF/Si coating, the incorporation of silane induced localized microphase separation, leading to interfacial defects within the otherwise homogeneous fluorocarbon matrix and thereby reducing the overall compactness of the coating. This microstructural feature provides direct experimental evidence for the slight reduction in gas-barrier efficiency of the composite coating and accounts for its intermediate carbonation resistance between the pure fluorocarbon coating and the uncoated control.

In summary, the incorporation of silane alters the compactness of the fluorocarbon film, thereby affecting the carbonation resistance of the overall protective system. Nevertheless, the hybrid structure formed by silane and fluorocarbon maintains the superior weathering resistance and chemical stability of the outer fluorocarbon film while markedly enhancing the system’s ability to resist chloride-ion ingress from both directions. Although the presence of silane slightly reduces the compactness of the outer fluorocarbon layer and consequently its carbonation resistance, the coating still satisfies the engineering specifications and exhibits outstanding comprehensive protective performance.

### 2.6. Comprehensive Performance Comparison and Analysis

The aforementioned findings indicate that the combination of silane and fluorocarbon resin forms a comprehensive protective system with complementary characteristics and synergistic effects, rather than a simple superposition of individual properties. This section aims to compare the performance of the composite coating system proposed in this study with representative existing protective systems by focusing on key indicators such as chloride ion penetration resistance and carbonation resistance, thereby further validating its protective advantages and application potential. The corresponding results are presented in Table 4 and Table 5.

Table 4 presents a comparison of the chloride migration coefficients of the protective systems developed in this study and those of commonly reported coating systems in the literature. SF/Si exhibits a reduction in chloride migration of 88.1% relative to the blank control, markedly surpassing conventional fluorocarbon (67.1%), polyurethane (47.8%), and acrylic resin (54.7%) coatings [50,51]. The corresponding chloride migration coefficient (4.9 × 10^−12^ m^2^/s) demonstrates excellent chloride resistance. Although SF/Si shows slightly lower chloride-ion protection than some silane systems, the enhancement in substrate protection achieved by SF/Si (88.1%) is comparable to that of the silane systems (84.3%) [52].

**Table 4:** Comparison of chloride ion resistance between the coatings studied and existing coatings.

Ref.	Coating Type	Test Method	Control/Coating (10^−12^ m^2^/s)	Reduction Rate (%)	Protective System Type
(SF/Si)	Fluorocarbon/Silane	RCM	41.1/4.9	88.1	inorganic-modified organic composite system
(F/Si)	Fluorocarbon	RCM	41.1/13.6	67.1	Organic system
[50]	Polyurethane	FT	9.0/4.7	47.8	Organic system
[51]	Acrylic	RCMT	14.4/6.53	54.7	Organic system
[52]	Nano-SiO_2_ modified silane	RCPT	20.93/3.29	84.3	Inorganic-modified system

Table 5 compares the carbonation depths of SF/Si, F/Si, and several representative protective systems after 28 days of accelerated carbonation. The results indicate that the combination of silane and fluorocarbon does not substantially diminish the improvement in carbonation resistance provided by fluorocarbon coatings, with reduction rates of 81.8% and 86.0% for SF/Si and F/Si, respectively. SF/Si therefore retains a relatively good carbonation resistance. When compared with certain polymer-based protective systems, such as acrylic emulsion coatings (carbonation depth: 2.98 mm; reduction: 70.8%) and nano-SiO_2_-modified polyurethane coatings (carbonation depth: 2.0 mm; reduction: 65.5%), SF/Si exhibits slightly weaker carbonation protection but still falls within the same protection grade, while offering a more pronounced overall improvement in performance [49,53,54]. In comparison, single silane coatings provide only limited enhancement in carbonation resistance, reflected by a reduction rate of approximately 13.6% [55].

**Table 5:** Comparison of carbonation resistance between the coatings studied and existing coatings.

Ref.	Coating Type	28d Carbonation Depth (mm)	Reduction Rate (%)	Protective System Type
(SF/Si)	Fluorocarbon/Silane	26.44/4.82	81.8	inorganic-modified organic composite system
(F/Si)	Fluorocarbon	26.44/3.71	86.0	Organic system
[53]	Acrylic	10.2/2.98	70.8	Organic system
[55]	Pure silane	23.6/20.4	13.6	Inorganic system
[54]	Nano-SiO_2_ modified Polyurethane	5.8/2.0	65.5	Organic-modified system

In summary, SF/Si, a novel polymer–silicate composite gel system, demonstrates excellent performance in two critical durability metrics: resistance to chloride ion penetration and carbonation. Compared with silane-based systems, SF/Si maintains comparable chloride-ion resistance while exhibiting a significantly enhanced carbonation resistance. When compared with polymer-based protective systems, SF/Si shows slightly lower carbonation resistance but still meets equivalent protection-grade standards, while its chloride-ion resistance is notably superior. These differences reflect the fundamental distinctions in protective mechanisms between different coating systems. Although silane-based systems can penetrate the substrate, they lack the intrinsic density and durability characteristic of polymer-based systems. Single polymer films, as discussed in Section 2.2, are unable to effectively prevent the back-diffusion of harmful ions from the concrete pores, thereby limiting long-term protective performance. SF/Si integrates the inherent density of the polymer film with the protective layer formed through silane penetration, creating a dual-layer synergistic system. This structure provides a balanced enhancement of durability and demonstrates improved overall protective performance.

## 3. Conclusions

This study developed a novel polymer–silicate composite gel system based on silane and fluorocarbon resin. Through the synergistic effect of dual-layer protection, the system significantly enhances mortar resistance to chloride penetration while maintaining inherent chemical durability. The main conclusions are summarized as follows:

1. The RCM test indicated that the SF/Si composite gel system had a chloride penetration depth of only 14.9 mm, representing a 57% reduction compared to the F/Si gel system. Its DRCM value was 4.91 × 10^−12^ m^2^/s, 63.97% lower than F/Si, demonstrating a more effective limitation of chloride ion migration.
2. Chloride natural diffusion tests demonstrated that after 14, 28, and 56 days of immersion, SF/Si contained less free chloride across the full depth compared to F/Si. Within the 0–5 mm range, SF/Si’s free chloride content was 0.121%, 0.225%, and 0.412%, corresponding to reductions of 55.35%, 50.10%, and 43.64% relative to F/Si. This indicates that the novel polymer–silicate composite gel system exhibits superior resistance to chloride penetration compared to the fluorocarbon–silicate gel system.
3. SEM images revealed that SF/Si exhibited fewer surface NaCl crystal precipitations and a slower hydration reaction, suggesting enhanced hydrophobicity. This effectively restricted chloride transport, allowing SF/Si to resist chloride penetration more efficiently than F/Si.
4. The incorporation of silane had minimal impact on the inherent acid and alkali resistance of the F/Si gel system, with SF/Si maintaining comparable performance to F/Si under acidic and alkaline conditions.
5. The incorporation of silane components decreased the compactness of the coating, resulting in a carbonation resistance slightly lower than that of the pure fluorocarbon coating. Nevertheless, according to the JGJ/T193-2009 standard, the 28-day carbonation depth of SF/Si (4.82 mm) still corresponds to Class T-IV, indicating a satisfactory engineering performance.

This study verified the effectiveness of the silane–fluorocarbon composite system in improving the chloride ion resistance of concrete, providing a feasible technical approach for enhancing the durability of concrete structures under harsh environments. From a lifecycle perspective, the fluorocarbon component, due to its excellent chemical stability and dense film-forming characteristics, can significantly extend the service life of structures and reduce maintenance frequency, which has positive significance for engineering sustainability. However, as a recalcitrant material, fluoropolymers still face certain controversies regarding their environmental sustainability. Therefore, under the premise of maintaining protective performance, further research on reducing the fluorocarbon content or using alternative green resins (such as waterborne acrylic and waterborne epoxy resins) is necessary for the effective application of the protective system in this study. At the same time, a comprehensive assessment of the lifecycle performance of the coating system, including durability, sustainability, and environmental impact, should be conducted to provide theoretical support for the development of future green concrete protective materials from the perspectives of service performance and ecological benefits.

## 4. Materials and Methods

### 4.1. Experimental Materials

The cement used in this study was ordinary Portland cement (P·O 42.5) produced by China Resources Cement Co., Ltd., China. River sand with a fineness modulus of 2.8, sourced from Xinyang, Henan, China, was used as the fine aggregate. A silane emulsion was supplied by Henan Juyan Co., Ltd., China. The fluorocarbon emulsion was prepared following the formulation provided by Henan Juyan Co., Ltd., with the main raw material composition summarized in Table 6. All water used in the experiments was obtained from the laboratory.

### 4.2. Preparation of Mortar Specimens

Following the guidelines outlined in the “Standard Test Method for Strength of Cement Mortar” (GB/T17971-2021) [56], two types of mortar specimens, Φ100 mm × 100 mm, 100 mm × 100 mm × 100 mm and 40 mm × 40 mm × 160 mm, were prepared and placed in a constant-temperature curing chamber for curing.

### 4.3. Preparation of Test Materials

**Figure 12:**
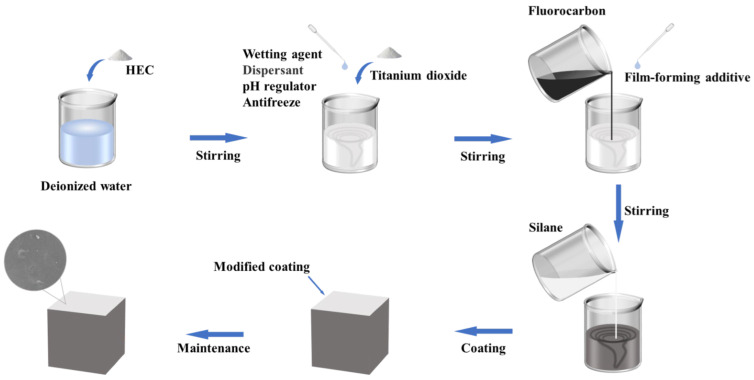
Preparation process of silane/fluorocarbon–silicate gel system.

The preparation process of the silane/fluorocarbon–silicate (SF/Si) gel system is illustrated in Figure 12: Deionized water was poured into a container, and a mixer was started. HEC was slowly added while stirring for 10 min to ensure full mixing with water. pH adjuster, dispersing agent, wetting agent, antifreezing agent, and titanium dioxide were sequentially added and stirred continuously for 40 min. Then, the film-forming aid and liquid fluoropolymer were added and stirred for another 10 min. Finally, a thickener, biocide, 0.3% carbon black pigment, and defoamer were added and stirred for 10 min to form a fluorocarbon emulsion. Silane emulsion and fluorocarbon emulsion were weighed at a mass ratio of 1:3 and thoroughly stirred for 3 min to obtain the silane-modified fluorocarbon coating. The resulting coating was evenly applied to the surface of cement mortar specimens to form the SF/Si gel system. For comparison, a fluorocarbon emulsion was applied to the specimens to prepare the fluorocarbon–silicate (F/Si) gel system.

### 4.4. Test Method

#### 4.4.1. RCM Test

Following the guidelines specified in the “Standard Test Method for Long-Term Performance and Durability of Ordinary Concrete” (GB/T50082-2009) [57], the rapid chloride migration (RCM) method was employed to determine the chloride penetration depth and migration coefficient in mortar. The specimens were placed in a vacuum saturation apparatus (VJH, Beijing Kangluda Experimental Instrument Co., Ltd., Beijing, China). Vacuum saturation was conducted at 0.013 MPa for 3 h, followed by a pressure saturation for 1 h and subsequent immersion for 18 h, resulting in a total saturation duration of 22 h. After saturation, the specimens were removed, surface moisture was wiped off, and the specimens were mounted on a rapid chloride migration (RCM) apparatus (RCM-6, Beijing Kangluda Experimental Instrument Co., Ltd., Beijing, China). The anode compartment was filled with 0.3 mol/L NaOH solution, while the cathode contained 10 wt% NaCl solution. An initial current was measured at 30 V to determine the appropriate voltage for the subsequent test. The applied voltage was then adjusted based on the measured initial current, and the test duration was determined accordingly.

#### 4.4.2. Chloride Ion Natural Erosion Test

The specimens with dimensions of 100 mm × 100 mm × 100 mm were immersed in a 3.5% NaCl solution for 14, 28, and 56 days, with the solution replaced every 7 days. After the designated immersion period, the specimens were removed, and surface residual moisture was wiped off. Powder samples were collected by drilling at the test surface, with sampling conducted every 5 mm up to a depth of 60 mm. After drying, the powder samples were analyzed using a rapid chloride ion content tester (CLU-H, Beijing Kangluda Experimental Instrument Co., Ltd., Beijing, China) to determine the free chloride ion content (mass fraction of chloride ions relative to the specimen powder).

#### 4.4.3. Acid and Alkali Corrosion Test

The 5% H_2_SO_4_ and 5% NaOH solutions were prepared, and mortar specimens with dimensions of 40 mm × 40 mm × 160 mm were immersed in the corresponding solutions. At 5, 10, 15, 20, 25, and 30 days of immersion, the specimens were removed, surface residues were cleaned, and the specimens were wiped dry. The visual condition and damage of each specimen were inspected, and the specimen mass was measured. Subsequently, the flexural strength was tested using a universal testing machine (DYE-600S-20, Wuxi Construction Testing Instrument Manufacturing Co., Ltd., Wuxi, China), and the two halves resulting from the flexural test were further used for compressive strength testing. A constant loading rate of 1500 N/s was applied until failure. The ultimate load at failure ($N_{u}$) was recorded, and the compressive strength was calculated using the conversion factor K=1.35, thereby converting the strength of non-standard specimens to the equivalent standard cubic compressive strength [58].

#### 4.4.4. Carbonation Test

The carbonation test was conducted according to the “Standard for Test Methods of Long-term Performance and Durability of Ordinary Concrete” GB/T 50082-2009 [57]. The test specimens included SF/Si, F/Si, and a blank control group, each in the form of 100 mm × 100 mm × 100 mm mortar cubes. All specimens were placed in a carbonation chamber (CCB-70, Beijing Kangluda Experimental Instrument Co., Ltd., Beijing, China) and subjected to accelerated carbonation under conditions of CO_2_ concentration of (20 ± 3)%, relative humidity of (70 ± 5)%, and temperature of (20 ± 2) °C.

### 4.5. Statistical Analysis

All quantitative experimental data were subjected to statistical analysis, following the procedures below:

(1) Data presentation

All data are presented as mean ± standard deviation, with the number of replicates for each experimental group n=3.

(2) Homogeneity of variance test

Prior to performing analysis of variance, Levene’s test was used to verify the homogeneity of variances. All datasets satisfied the homogeneity assumption (p>0.05).

(3) Significance tests

One-way analysis of variance (one-way ANOVA) was used to compare the differences in chloride migration coefficients among the control group, the fluorocarbon-coated group, and the silane-fluorocarbon composite-coated group.

Two-way analysis of variance (two-way ANOVA) was employed to compare differences in free chloride content, mass loss, and compressive strength loss under acid-base exposure among the same three groups.

Post-hoc Tukey HSD tests were performed to compare the specific differences between the two coating systems and the control group under each experimental condition, evaluating the extent to which the coatings improved mortar performance.

All statistical analyses were performed using Excel 2021 (Microsoft Corp., Redmond, WA, USA), along with SPSS Statistics 26.0 (IBM Corp., Armonk, NY, USA) for the ANOVA and post-hoc tests.

(4) Significance level

The significance level was set at α=0.05, and p<0.05. was considered statistically significant. The raw experimental data are provided in the Supplementary Materials.

## Figures and Tables

**Table 1:** Comparison of chloride migration coefficients of different materials.

Systems Type	Depth of Penetration (mm)	D_RCM_ (10^−12^ m^2^/s)	D_RCM_ Decline Rate (%)
blank	44.2 ± 1.1	41.1 ± 1.8	--
SF/Si	14.8 ± 0.8	4.9 ± 0.2	88.1
F/Si	35.0 ± 1.5	13.6 ± 0.4	67.1

Notes: Data are presented as mean ± standard deviation (SD), *n* = 3.

**Table 2:** Ionic radius and crystalline cell parameter data (pm).

Chloride Ion Radius	Calcium Ion Radius	Sodium Ion Radius	Carbonate Ion Radius	CaCO_3_ Unit Cell Parameters a	NaCl Unit Cell Parameters a
181	100	102	178	499	564

**Table 3:** Classification of concrete carbonation resistance levels.

Grade	T-I	T-II	T-III	T-IV	T-V
Carbonation Depth d (mm)	d ≥ 30	20 ≤ d < 30	10 ≤ d < 20	0.1 ≤ d < 10	d < 0.1

**Table 6:** Fluorocarbon component main raw material ratio.

Name	Dosage (g)	Mass Fraction (%)
Deionized water	90	30
Hydroxyethyl cellulose	0.75	0.25
Dispersant	1.8	0.6
pH adjuster	0.45	0.15
Wetting agent	0.4	0.13
Antifreeze agent	3	1
Titanium dioxide R-699	60	20
Film-forming additive	11	3.67
Liquid Fluorocarbon	135	45

## Data Availability

The original contributions presented in this study are included in the article. Further inquiries can be directed to the corresponding author.

## Supplementary Materials

The following supporting information can be downloaded at: https://www.mdpi.com/article/10.3390/gels11120936/s1, Table S1: Levene’s Test and ANOVA results. Table S2: Post Hoc test.
